# Editorial

**DOI:** 10.1093/emph/eot017

**Published:** 2013-09-11

**Authors:** Stephen C. Stearns

**Affiliations:** Editor-in-Chief, *Evolution, Medicine, and Public Health*, New Haven, CT

In our first year of publication, my main concern has been to encourage the submission, competent and critical review, and rapid publication of substantial science that will enhance the intellectual reputation of evolutionary approaches to medicine and public health. In that effort, we have been very well supported by OUP.

First a few numbers:

- We began accepting manuscripts in July 2012; our first volume is 2013.
- We have been accepting about 40% of the manuscripts submitted.
- The average number of days from submission to first decision is 20, to final decision, 39.
- At the time this was written, we had published 10 original research articles, 3 review articles, 2 commentaries, 2 interpretive essays, 1 brevia, 1 book review and 1 editorial. That balance is about what we are aiming for.
- Readers have downloaded PDFs of *EMPH* articles over 1700 times.

The numbers document moderate growth and fairly rapid publication of a balanced mix of article types.

*EMPH* is attracting some notice. While we are not yet listed on PubMed (one must first have published 40 articles that have been cited), that should come within the next year. In the meantime, Google Scholar easily finds articles in *EMPH*.

A tip to prospective authors—I usually reject two types of manuscripts without review: papers that contain speculative arguments from evolutionary psychology used to support a favoured hypothesis without testing alternative explanations, and papers that document some phenomenon—such as the evolution of antibiotic resistance—that is already well understood, i.e. competent pieces of research that add nothing to our general knowledge. Authors of such papers could save time and effort by submitting elsewhere.

I encourage readers of this editorial to submit some of their good, appropriate work to *EMPH*. I do not expect submissions that you plan to send to a high-impact, general-interest journal, but if that great paper does not get into the high-impact journal to which you first submit it, send it to *EMPH* for your next try. We are especially interested in papers with evolutionary insights into: cancer; alternatives to traditional antibiotic therapy; the costs of inflammation; virulence, tolerance and resistance; reproduction; the microbiome, atopies, autoimmune disease and obesity; and the medical curriculum.

